# Supplementary material for: One in five South Africans are multimorbid: An analysis of the 2016 demographic and health survey
Source: PLoS One. 2022 May 26;17(5):e0269081. doi: 10.1371/journal.pone.0269081 (PMC9135225; doi:10.1371/journal.pone.0269081)
Supplement: S1 File — (DOCX) [file pone.0269081.s001.docx]

**Supporting information**

S1 Table provides details on the self-reported variables included in this study. It provides the survey question used, notes on data cleaning and the coding utilised for the variables of interest.

**S1 Table. Coding for self-reported disease conditions, sociodemographic details and behavioural factors**

|  | **Variable** | **Survey Question** | **Notes on data cleaning** | **Coding** |
| --- | --- | --- | --- | --- |
| **Disease conditions** | **Diabetes** | Has a doctor, nurse or health worker told you that you have or have had any of the following conditions: diabetes or blood sugar? | None. | - No=0 - Yes=1 |
|  | **Emphysema/ Bronchitis/COPD** | Has a doctor, nurse or health worker told you that you have or have had any of the following conditions: chronic bronchitis, emphysema, or COPD? | None. | - No=0 - Yes=1 |
|  | **Heart disease** | Has a doctor, nurse or health worker told you that you have or have had any of the following conditions: Heart attack or angina/chest pains? | None. | - No=0 - Yes=1 |
|  | **High blood cholesterol** | Has a doctor, nurse or health worker told you that you have or have had any of the following conditions: high blood cholesterol or fats in the blood? | None. | - No=0 - Yes=1 |
|  | **Stroke** | Has a doctor, nurse or health worker told you that you have or have had any of the following conditions: stroke? | None. | - No=0 - Yes=1 |
|  | **TB in the last 12 months** | Has a doctor, nurse or health worker ever told you that you have TB? | Two questions were combined. ‘No’ refers to people who have not had TB or had TB more than 12 months ago. ‘Yes’ refers to people who had TB in the last 12 months. | - No=0 - Yes=1 (TB in the last 12 months) |
|  |  | When was the last time you had TB? |  |  |
| **Sociodemographic details** | **Educational attainment** | What is the highest grade or form you completed at that level? | Responses were divided into three categories. | - Primary or less=0 - Secondary complete=1 - Tertiary=2 |
|  | **Wealth index** | *Various questions.* | The 2016 SADHS wealth index was used. The wealth index scores households according to the types of goods that are owned and other characteristics; and uses a principal component analysis to score the households [[20](#_ENREF_20)]. | - Quintile 1 (Poorest)=0 - Quintile 2 (Poorer)=1 - Quintile 3 (Middle)=3 - Quintile 4 (Richer)=4 - Quintile 5 (Richest)=5 |
| **Behaviour** | **Current alcohol use** | Have you ever consumed a drink that contains alcohol such as beer, wine, ciders, spirits, or sorghum beer? | Responses to the two questions were combined. “Yes” refers to participants who drank alcohol in the past 12 months and “No” refers to participants who have not drank alcohol or have drank more than 12 months ago. | - No=0 - Yes=1 |
|  |  | Was this within the last 12 months? |  |  |
|  | **Current tobacco smoker** | Do you currently smoke tobacco every day, some days, or not at all? | “No” represents participants who do not smoke. “Yes” represents participants who smoke daily or occasionally. | - No=0 - Yes=1 |

S2 Table provides additional information regarding the collection of physically measured variables included in the study. It also supplies information regarding the cleaning procedure and coding of the variables of interest. For diabetes specifically, there was a self-reported variable and a biomarker variable which were combined to create a new variable.

**S2 Table: Description of data collection and data cleaning.**

| **Variable** | **Description of data collection** | **Data cleaning** | **Coding** |
| --- | --- | --- | --- |
| **Diabetes (HbA1c)** | For participants over age 15 years who consented to HbA1c testing, nurses collected blood specimens from finger pricks. If the participant consented to both HbA1c and HIV testing, five blood spots were collected on filter paper card and a unique barcode was affixed to the card.  The dry blood specimens (DBS) were analysed with a blood chemistry analyser which measured total haemoglobin concentration by a colorimetric method.  A more detailed description can be found in the SADHS 2016 Report, p.5 [[20](#_ENREF_20)]. | The data cleaning followed the procedure used for the diabetes risk factor in the second South African Comparative Risk Assessment Study [[25](#_ENREF_25)]. Inconclusive HbA1c values were eliminated.  An adjustment for HbA1c DBS was made, as detailed in SADHS 2016 Report, p 271.  Diabetic status was assigned if HbA1c >=6.5 mmol.  Those on diabetes medication were added to people with diabetes. | - No= 0 - Yes=1 (has diabetes) |
| **Self-reported diabetes / biomarker diabetes** |  | The results of the self-reported diabetes and HbA1c diabetes were combined. | Self-reported diabetes / biomarker diabetes:   - No= 0 - Yes=1 (has diabetes) |
| **HIV** | If the participant consented to both HbA1c and HIV testing, five blood spots were collected on filter paper card and a unique barcode was affixed to the card.  All samples were tested with an enzyme-linked immunosorbent assay (ELISA), the Genscreen HIV 1/2 Combi Assay (Bio-Rad). If the first ELISA was positive, a second ELISA was done (ELISA 2), the E411 Cobas HIV 1/2 Combi Assay (Roche).  A more detailed description can be found in the SADHS 2016 Report, p.5 [[20](#_ENREF_20)] | The first HIV assay result was taken as confirmation of HIV. | - No=0 - Yes=1 (has HIV) |
| **Anaemia** | For participants over age 15 years who consented to anaemia testing, nurses collected blood samples were drawn from a finger prick and collected in a microcuvette.  The analysis of haemoglobin was conducted on site using a battery-operated portable HemoCue 201+ analyser. Nonpregnant women, pregnant women, and men were referred if their haemoglobin levels were below 7 g/dl, 9 g/dl, and 9 g/dl, respectively. (SADHS 2016 Report, p. 5).  The SADHS 2016 anaemia results were adjusted for smoking status and altitude. | Anaemia was coded in the DHS 2016 as: None, mild, moderate and severe.  According to the DHS-7 Standard Recode Manual [[23](#_ENREF_23)], anaemia levels below 7.0 g/dl are considered as severe anaemia. Moderate anaemia is considered levels between 7.0g/dl and 9.9g/dl. For pregnant women, mild anaemia are levels between 10.0 g/dl and 10.9 g/dl and between 10.0 g/dl and 11.9 g/dl for all other adult women.  These categories were recoded to anaemia present (mild, moderate, severe) or absent. | - No=0 - Yes=1 (has mild, moderate or severe anaemia) |
| **Hypertension** | For participants who consented to having their blood pressure measurements taken, three readings were taken using Omron 1300 digital blood pressure monitors. These measurements were taken at three-minute intervals.  The 2016 SADHS took the third measurement to classify the participant with hypertension according to the WHO 1999 categories.  A more detailed description can be found in the SADHS 2016 Report, p.5 [[20](#_ENREF_20)] | The data cleaning followed the procedure used for the hypertension risk factor in the second South African Comparative Risk Assessment Study[[26](#_ENREF_26)]. The data cleaning and recording procedure was in agreement with the procedure applied by [Zhou, Bentham, Di Cesare, Bixby, Danaei, Cowanet al. (46)](#_ENREF_46).  Excluding the first, the average of the remaining replicated readings was considered as the subject blood pressure. If one value was missing of the two readings, the observation was excluded.  Replicated measurements of systolic and diastolic blood pressure were assessed for the presence of implausible values (systolic BP < 70 mm Hg or > 270 mm Hg, diastolic BP < 30 mm Hg or > 150 mm Hg), which were set to missing.  Hypertension categories are based on [Chobanian, Bakris (24)](#_ENREF_24). Categories include:   - Hypertension absent:   Systolic < 120 *mmHg* & diastolic < 80 *mmHg*   - Pre-hypertension:   Systolic: 120–139 *mmHg* or diastolic: 80-89 *mmHg*   - Stage 1 Hypertension:   Systolic: 140–159 *mmHg* or diastolic: 90–99 *mmHg*   - Stage 2 hypertension   Systolic ≥160 *mmHg* or diastolic ≥100 *mmHg*  People on medication were included in those that had hypertension. Hypertension was coded as absent or present (stage 1 or stage 2 hypertension). | Hypertension:   - No=0 - Yes=1 (has hypertension stage 1 or 2)   Hypertension categories:   - Normal=0 - Pre-hypertension=1 - Stage 1 hypertension =2 - Stage 2 hypertension=3 |
| **Body Mass Index (BMI)** | For participants over the age of 15 years who consented to having their height and weight measured, a Seca 878 digital scale and Seca 213 portable stadiometer was used.    A more detailed description can be found in the SADHS 2016 Report [[20](#_ENREF_20)] | The data cleaning followed the procedure used for the BMI risk factor in the second South African Comparative Risk Assessment Study [[27](#_ENREF_27)]. Implausible values were converted to missing values. Height in centimetres was calculated. The BMI was calculated using the *BMI* STATA package.  **BMI category BMI (kg/m2) range**   - Underweight 15 .0 - <18.5 - Normal weight 18.5 - <25.0 - Overweight 25.0 - <30.0 - Obesity grade 1 30.0 - <35.0 - Obesity grade 2 35.0 - <40.0 - Obesity grade 3 40.0 - <60.0 | BMI categories:   - Underweight=0 - Normal weight=1 - Overweight=2 - Obesity grade 1=3 - Obesity grade 2=4 - Obesity grade 3=4 |

The unweighted prevalence of multimorbidity is shown in S3 Table.

**S3 Table. Prevalence of multimorbidity (unweighted data).**

|  | **Unweighted** | | |
| --- | --- | --- | --- |
| **Number of diseases** | **Total**  **% (n= 10 336)** | **Male**  **% (n= 4 210)** | **Female**  **% (n= 6 126)** |
| No disease | 45.0 | 52.8 | 39.7 |
| 1 disease | 32.1 | 31.2 | 32.7 |
| 2 diseases | 16.0 | 11.6 | 19.1 |
| 3 diseases | 5.4 | 3.6 | 6.7 |
| 4 diseases | 1.2 | 0.7 | 1.5 |
| 5 diseases | 0.2 | 0.1 | 0.4 |
| 6 diseases | 0.06 | 0.02 | 0.1 |
| **Multimorbidity (≥ 2 diseases)** | **22.9** | **16.0** | **27.7** |

S4 Table gives the weighted prevalence of multimorbidity by age group and sex.

**S4 Table. Multimorbidity prevalence by age group and sex.**

| **Age group (years)** | **Multimorbidity prevalence** | | |
| --- | --- | --- | --- |
|  | **Total**  **% (95% CI)** | **Males**  **% (95% CI)** | **Females**  **% (95% CI)** |
| 15-19 | 3.0  (2.2 - 4.1) | 1.9  (1.1 - 3) | 4.4  (3 - 6.5) |
| 20-29 | 9.8  (8.3-11.5) | 4.8  (3.5 - 6.6) | 14.9  (12.3 - 17.8) |
| 30-39 | 18.0  (15.6-20.5) | 11.2  (8.2 - 15.1) | 24.6  (21.5 - 28) |
| 40-49 | 24.7  (22-27.6) | 18.5  (14.8 - 22.8) | 30.2  (26.7 - 34) |
| 50-59 | 35.8  (32.7-39.1) | 31.9  (26.8 - 37.5) | 38.6  (34.7 - 42.8) |
| 60-69 | 42.2  (37.9-46.7) | 36.6  (30.2 - 43.6) | 47.2  (42.3 - 52.1) |
| 70-79 | 42.9  (36.3-49.6) | 40.1  (31.4 - 49.5) | 44.6  (37 - 52.4) |
| 80+ | 41.9  (34.5-49.6) | 38.6  (23.8 - 55.7) | 43.7  (35.9 - 52) |

S5 Table shows the results of additional models (Models 1 and 2),

**S5 Table. Factors associated with multimorbidity**

| **Variable** | **Unadjusted**  **Odds ratios**  **(95% CI)** | **Model 1**  **Odds Ratio**  **(95% CI)** | **Model 2**  **Odds ratio**  **(95% CI)** |
| --- | --- | --- | --- |
| Age category *(Reference: 15 – 24 year)* | | | |
| - 25 - 34 years | **2.982 (2.407 - 3.695) *** | **2.902 (2.342 - 3.595) *** | **3.262 (2.603 – 4.088) *** |
| - 35 - 44 years | **4.861 (3.769 - 6.269) *** | **4.803 (3.728 - 6.188) *** | **5.540 (4.286 – 7.160) *** |
| - 45 – 54 years | **7.527 (5.844 - 9.694) *** | **7.208 (5.602 - 9.274) *** | **8.334 (6.422 - 10.814) *** |
| - 55 – 64 years | **11.764 (8.837 - 15.662) *** | **11.412 (8.553 - 15.225) *** | **12.549 (9.362 - 16.821) *** |
| - 65+ years | **14.181 (10.951 – 18.364) *** | **13.530 (10.462 - 17.499) *** | **13.924 (10.597 - 18.294) *** |
|  |  |  |  |
| Sex (*Reference: Male)* | **2.038 (1.804 – 2.301) *** | **1.902 (1.672 – 2.163) *** | **1.832 (1.606 - 2.090) *** |
|  |  |  |  |
| - 15-24#Female | - | - | - |
| - 25-34#Female | - | - | - |
| - 35-44#Female | - | - | - |
| - 45-54#Female | - | - | - |
| - 55-64#Female | - | - | - |
| - 65+#Female | - | - | - |
|  |  |  |  |
| Urban *(Reference: Rural)* | **0.817 (0.721 – 0.925) *** | - | 0.939 (0.774 - 1.138) |
|  |  |  |  |
| Education *(Reference: Primary)* | | | |
| - Secondary | **0.423 (0.372 -0.480) *** | - | 0.944 (0.812 - 1.097) |
| - Tertiary | **0.323 (0.251 – 0.414) *** | - | **0.640 (0.483 - 0.848) *** |
|  |  |  |  |
| Wealth index (Reference: Poorest) | | | |
| - Poorer | 0.995 (0.829 - 1.194) | - | 1.041 (0.847 - 1.281) |
| - Middle | 1.076 (0.874 - 1.324) | - | 1.152 (0.898 - 1.479) |
| - Richer | 1.036 (0.845 - 1.270) | - | 1.083 (0.821 - 1.428) |
| - Richest | 0.901 (0.713 – 1.138) | - | 0.788 (0.574 - 1.081) |
|  |  |  |  |
| Employed *(Reference: Not employed)* | **0.744 (0.643 – 0.861) *** | - | **0.779 (0.659 - 0.922)*** |

S1 Fig shows the standardised Pearson residuals against the inverse normal to test for the fit of final model (Model 3).

**S1 Fig. Final model standardised Pearson residuals against the inverse normal.**

S2 Fig shows the standardised Pearson residuals for the final model (Model 3).

**S2 Figure. Final model standardised Pearson residuals.**

S3 fig displays the hypertension prevalence in people with HIV by age group (weighted).

**S3 Figure.** **Hypertension prevalence in people living with HIV (weighted).**
