# Supplementary material for: One in five South Africans are multimorbid: An analysis of the 2016 demographic and health survey
Source: PLoS One. 2022 May 26;17(5):e0269081. doi: 10.1371/journal.pone.0269081 (PMC9135225; doi:10.1371/journal.pone.0269081)
Supplement: Attachment — Submitted filename: One in five_SADHS2016_manuscript_Edited_Kassa_Dec2021.docx [file pone.0269081.s002.docx]

One in five South Africans are multimorbid: An analysis of the 2016 Demographic and Health Survey

Rifqah Abeeda Roomaney,^1 2^ Brian van Wyk ,^2^ Annibale Cois,^1 3^ Victoria Pillay van Wyk ^1^

^1^ Burden of Disease Research Unit, South African Medical Research Council, Cape Town, South Africa

^2^ School of Public Health, University of the Western Cape, Cape Town, South Africa

^3^ Division of Health Systems and Public Health, Department of Global Health, University of Stellenbosch, South Africa

*Corresponding author:

Email: [rifqah.roomaney@mrc.ac.za](mailto:rifqah.roomaney@mrc.ac.za) (RAR)

**ORCID IDs:**

Rifqah A Roomaney <http://orcid.org/0000-0003-3267-8484>

Brian van Wyk <http://orcid.org/0000-0003-1032-1847>

Annibale Cois <https://orcid.org/0000-0002-7014-6510>

Victoria Pillay-van Wykhttp://orcid.org/0000-0001-8772-7197Abstract

Multimorbidity is a global research priority, yet relatively little is known about it in low and middle income countries. South Africa has the largest burden of HIV worldwide but also has a growing burden of non-communicable diseases; potentially leading to uncommon disease combinations. Information about the prevalence of multimorbidity and factors associated with it can assist in healthcare planning and targeting groups of people for interventions. This study aimed to determine the prevalence of multimorbidity by age and sex, as well as factors associated with multimorbidity in people over the age of 15 years. This study analyses the nationally representative 2016 South African Demographic Health Survey. The sample included 10 336 people who participated in the Adult Health questionnaire and approximately 7 961 people who provided biomarkers. Multivariate logistic regression was used to measure the association of multimorbidity with age, sex, living in an urban or rural area, education level, wealth level, employment status, body mass index, current alcohol or tobacco use. All analyses were conducted using STATA 15. Multimorbidity was present in 20.7% (95% CI: 19.5% – 21.9%) of participants; in 14.8% (95% CI: 13.4% - 16.3%) of males and 26.2% (95% CI: 24.7 – 27.7%) of females. Multimorbidity increased with age; being most common in the 55 - 64 years old age group (OR: 24.910, 95% CI: 14.901 - 41.641, p < 0.001) compared to those aged 15 – 24 years. The odds of multimorbidity was also higher in young females compared to young males (OR: 2.734, 95% CI: 1.50 – 4.99, p= 0.001). Possessing tertiary education (OR: 0.722, 95% CI: 0.537 - 0.97, p=0.031), being employed (OR: 0.813, 95% CI: 0.675 - 0.979, p=0.029) or currently using alcohol (OR: 0.815, 95% CI: 0.686 – 0.968, p=0.02) was protective against multimorbidity. Multimorbidity is prevalent within the South African population, with females and older adults being most affected. However, multimorbidity is also observed in younger adults and most likely driven by the high prevalence of HIV and hypertension.

# Introduction

People living with more than one disease (also known as multimorbidity) have their lives impacted in many ways; including through having a reduced quality of life [1-6], an increased risk of dying [7-9] and an intensified need to utilise healthcare [10-14]. Despite these negative impacts, the area of multimorbidity remains under researched when compared to research afforded to single disease conditions [15]. This is particularly acute in low and middle income countries (LMICs) where 5% of multimorbidity research globally has taken place [15]. Little is known about multimorbidity in LMICs where disease burdens are thought to differ from countries with more established multimorbidity profiles.

South Africa is an upper middle-income country [16] with a quadruple burden of disease consisting of: HIV/AIDS and tuberculosis (TB); other communicable diseases, perinatal conditions, maternal causes, and nutritional deficiencies; non-communicable diseases (NCDs); and injuries [17]. South Africa has a very high HIV prevalence and it is not uncommon that people living with HIV also develop other chronic conditions. Given this HIV burden, information is needed on the prevalence of multimorbidity to plan for more responsive healthcare services. This information is valuable for planning purposes as health service delivery could be made more efficient around common disease clusters to the benefit of those living with multimorbidity. Knowing who is most affected by multimorbidity (i.e. determinants or factors that are common in those affected) can also be used to design interventions to target those individuals. While multimorbidity research has been emerging in the country for the past decade, few studies have reported the prevalence of multimorbidity and factors associated with it in a consistent and comparable manner [18].

National survey data is an important source of information about multimorbidity. National surveys represent a largely untapped resource that could shed light on multimorbidity in the general population. This is especially true for LMICs such as South Africa where limited information exists about multimorbidity. The Demographic and Health Surveys (DHS) project, primarily funded by the United States Agency for International Development has conducted more than 230 nationally representative comparable household surveys in more than 80 countries since 1984 [19]. The DHS collects data on a range of topics such as fertility, contraception, maternal and child health, HIV, malaria and domestic violence [19]. For many countries, the DHS is an important source of information for policy making, monitoring and evaluation and as the country’s public health evidence base [19]. In terms of multimorbidity, the DHS collects information on self-reported health conditions and biological markers.

Three DHS have been conducted in South Africa to date, with the most recent one in 2016 (SADHS 2016). This paper reports the process and results derived from a systematic analysis of the SADHS 2016 to establish the prevalence and factors associated with multimorbidity in the South African population.

# Materials and methods

## Sample and data source

This paper presents a secondary analysis of national survey data from the SADHS 2016. The survey is nationally representative with the aim of providing up-to-date estimates of demographic and health indicators such as information on fertility levels, marriage, sexual activity, contraceptive use, nutrition, child mortality, aspects of child health, exposure to the risk of HIV infection, behaviour and health indicators [20]. The SADHS 2016 also collected information on anthropometry, anaemia, hypertension, HbA1c levels and HIV among adults 15 years and older.

The SADHS 2016 followed a stratified two-stage sample design and a total of 750 primary sampling units (PSUs) were selected and stratified by urban, traditional and farm areas. A fixed number of twenty dwelling units were randomly selected in each PSU. Of these dwelling units, sub-sampling occurred whereby half of the households were eligible for a South African-specific module on Adult Health that included the collection of biomarkers [20].

## Description of included variables

### Self-reported diseases

The study sample consisted of 10 336 people aged 15 years and older who completed the Adult Health module and were asked about the presence of several diseases (Table 1) e.g. “Has a doctor, nurse or health worker told you that you have or have had any of the following conditions”. The response to the questions were “No”, “Yes” or “Don’t know”, with the “Don’t know” response recorded as missing values. TB in the last 12 months was constructed from two other variables: whether a participant had ever had the disease and whether they had the disease in the last 12 months or more than 12 months ago.

The analysis included data where the variables were deemed to be “current” conditions. Disease conditions were excluded for the following reasons: (i) disease conditions that could not be assumed to be current at the time of the survey due to the way that the question was asked (ii) disease conditions that were considered to be acute or of short duration (iii) disabilities or injuries. Two clinicians assisted where the information was unclear. Further details are available in S1 Table.

**Table 1. Survey questions on self-reported diseases.**

| **Variable** | **Survey Question** |
| --- | --- |
| **Diabetes** | Has a doctor, nurse or health worker told you that you have or have had any of the following conditions: diabetes or blood sugar? |
| **Emphysema/ Bronchitis/COPD** | Has a doctor, nurse or health worker told you that you have or have had any of the following conditions: chronic bronchitis, emphysema, or COPD? |
| **Heart disease** | Has a doctor, nurse or health worker told you that you have or have had any of the following conditions: Heart attack or angina/chest pains? |
| **High blood cholesterol** | Has a doctor, nurse or health worker told you that you have or have had any of the following conditions: high blood cholesterol or fats in the blood? |
| **Stroke** | Has a doctor, nurse or health worker told you that you have or have had any of the following conditions: stroke? |
| **TB in the last 12 months** | Has a doctor, nurse or health worker ever told you that you have TB? |
|  | When was the last time you had TB? |

### Physically measured diseases (biomarkers)

Of the people included, approximately 74.4% (n= 7 961) of people also had information on physically measured diseases. The following information was of interest to the analysis: diabetes (HbA1c), HIV status (dry blood spot), blood pressure measurements, anaemia (Hb), anthropometry (height and weight). Nurses collected blood specimens from finger pricks.

For diabetes, dry blood spots were analysed using a blood chemistry analyser which measures total haemoglobin concentrations [20]. A participant was assigned diabetic status if their HbA1c >=6.5 mmol [21,22]. Participants with normal HbA1c values but on medication to manage diabetes were also assigned diabetic status. In addition, self-reported diabetes information was combined for participants without biomarker data.

For HIV, dry blood spots were tested with an enzyme-linked immunosorbent assay (ELISA) and a second ELISA was done for confirmation [20]. We included the results of the first ELISA in study.

For anaemia, nurses collected blood samples in a microcuvette and the analysis of haemoglobin was conducted on site. The SADHS 2016 anaemia results were adjusted for smoking status and altitude [20]. Anaemia levels below 7.0 g/dl were considered as severe anaemia. Moderate anaemia was considered levels between 7.0g/dl and 9.9g/dl. For pregnant women, mild anaemia were levels between 10.0 g/dl and 10.9 g/dl and between 10.0 g/dl and 11.9 g/dl for all other adult women [23]. We then recategorized participants into either having no anaemia or having anaemia.

Three blood pressure measurements were taken from participants using digital blood pressure monitors [20]. For this study, we excluded the first measurement and took the average of the remaining replicated measurements. The values were categorised as: hypertension absent (Systolic < 120 mmHg & diastolic < 80 mmHg), Pre-hypertension (Systolic: 120–139 *mmHg* or diastolic: 80-89 *mmHg*), Stage 1 Hypertension: (Systolic: 140–159 *mmHg* or diastolic: 90–99 *mmHg*)*,* Stage 2 hypertension (Systolic ≥160 *mmHg* or diastolic ≥100 *mmHg*) [24]. Hypertension was coded as being absent (normal or pre-hypertension) or present (Stage 1 or Stage 2 hypertension). People on medication to manage hypertension were included in those that had hypertension. Data cleaning for diabetes [25] and hypertension [26] followed the procedures used in the Second South African Comparative Risk Assessment. Further details of how data was collected, cleaned and coded is listed in S2 Table.

### Other variables of interest

Several other variables were also included in the analysis. This study made use of the SADHS 2016 wealth index. The wealth index uses principal component analysis to score households according to the types of goods that are owned and other characteristics [20]. The households were divided into five quintiles, from poorest (Quintile 1) to richest (Quintile 5). Educational attainment was also included and described by the ‘highest grade or form you completed at that level’. This study divided the responses into three categories: primary school or less, secondary school, and tertiary education.

This study also examined current alcohol and tobacco use. For current alcohol use, the responses to the following two questions were combined: *“Have you ever consumed a drink that contains alcohol such as beer, wine, ciders, spirits, or sorghum beer?”* and “*Was this within the last 12 months?*”. For tobacco use, the question “*Do you currently smoke tobacco every day, some days, or not at all?*”. Both variables were coded as binary.

The BMI of participants were also examined. Height and weight were measured using a digital scale and stadiometer [20]. BMI was calculated using the *BMI* STATA package. BMI was categorized as follows: underweight (15 .0 - <18.5 kg/m^2^), normal weight (18.5 - <25.0 kg/m^2^), overweight (25.0 - <30.0 kg/m^2^), obesity grade 1 (30.0 - <35.0 kg/m^2^), obesity grade 2 (35.0 - <40.0 kg/m^2^), obesity grade 3 (40.0 - <60.0 kg/m^2^).[27]. Data cleaning was done in accordance to another study [27]. Further details are listed in S2 Table.

## Analysis

The statistical analysis was done using STATA 15.0 (Stata Corporation, College Station, Texas, USA) software. The STATA survey set (‘*svy*’) of commands were used to account for the complex survey design. Sampling weights were calibrated against the Statistics South Africa mid-year population estimates [28].

### Distribution of multimorbidity in the population

Multimorbidity is frequently measured by counting the number of co-existing conditions, using a predefined list of medical conditions [29,30]. Various studies have used this technique when doing secondary data analysis. Our Estimation of multimorbidity included: self-reported diseases (e.g. bronchitis/ COPD, heart disease, high blood cholesterol, stroke, TB in the last 12 months), biomarker disease (e.g. HIV, anaemia, high blood pressure) and a combination of the two (i.e. diabetes). Disease variables were coded as binary (disease absent ‘0’ or disease present ‘1’). An index variable was created where for each individual, the number of disease conditions present was counted. If there was information about a disease condition missing, this was counted as “no disease present”. The disease index variable was further categorised to create another variable, the Multimorbidity Index. This variable categorised individuals into either having “no multimorbidity” (no disease or only one disease present) or “multimorbidity present” (two diseases or more present).

For unweighted data (sample), frequencies were used to display categorical data. Age was analysed as a continuous variable while gender, locality, province, educational level and wealth index were analysed as categorical variables. Bivariate associations between locality, province, highest education level and wealth index by sex were assessed using Chi-square tests. The prevalence of having single disease conditions by sex was also assessed with Chi-square tests. For weighted data, multimorbidity status was described using histograms and box plots against age.

### Factors associated with multimorbidity

Regression methods were used to describe the relationship between a dependent variable and other predictor variables.[31] In this case, a multivariate logistic regression was employed because the dependent variable was binary *(Multimorbidity absent = 0, Multimorbidity present =1)*. Three models were constructed for logistic regression with multimorbidity as the dependent variable.

Systematic reviews identified the following characteristics (among others) as being related to multimorbidity: (i) Biomedical and individual: ageing, female, (ii) Socioeconomic: lower socioeconomic status, high-income group (in low and middle-income countries), lower education, (iii) Social and environmental: living in urban environments (iv) Behavioural: tobacco, overweight and obese [32]. For this study, the following variables were investigated as predictor variables: age category, sex, locality, highest education level, wealth index*,* employment status, BMI category, current smoker status and current alcohol drinker status.

Crude odds ratios were estimated by only including the dependent variable and one predictor variable. Model 1 contained only demographic information (e.g. age and sex), while Model 2 contained sociodemographic information (e.g. age, sex, educational attainment, wealth index and employment status). The final model (Model 3) included all variables in the previous models but also included lifestyle or behavioural factors (e.g. alcohol use, tobacco use and BMI).

Model checking was performed using various statistical tests. The link test [33] was used to determine if there were specification errors. Interaction terms were added where necessary. We Influential observations were checked using the Pearson residuals, deviance residuals and Pregibon leverage [34] on the unweighted model as these tests cannot be used on survey weighted data. Influential observations were dropped, and the model was refitted. The crude and adjusted odds ratios were reported with 95% CIs and *p*-values of less than 0.05 were considered as statistically significant.

# RESULTS

## Sample description

There were 10 336 males and females over the age of 15 included in the sample; with more females (59.2%) than males (Table 2). The median age of participants was 36 years (interquartile range: 24 – 52 years), with females being slightly older than males but this was not statistically significant. More than half of the sample resided in urban areas (55.0%) and most (64.5%) completed secondary education. The majority of participants were Black African (84.7%), followed by coloured (9.6%), white (4.4%) and Indian/Asian (1.4%). Age, urban location and education did not differ between males and females. There were significant differences in participation between males and females by province (p < 0.001) and wealth quintile (p = 0.018**).**

**Table 2. Description of sample population (unweighted).**

|  | Total (N=10 336)  % (n) | Male (N=4210)  % (n) | Female (N= 6126)  % (n) | *p*-value’ |
| --- | --- | --- | --- | --- |
| Age* (Median years and IQR) | 36 (24 – 52) | 33 (22 - 49) | 37 (25 – 54) | 0.442 |
| Urban location | 55.0 (5 685) | 55.2 (2 324) | 54.86 (3 361) | 0.735 |
| Province: |  |  |  | **<0.001** |
| - Western Cape | 7.29 (754) | 6.65 (280) | 7.74 (474) |  |
| - Eastern Cape | 13.08 (1 352) | 13.16 (554) | 13.03 (798) |  |
| - Northern Cape | 8.53 (882) | 8.38 (353) | 8.64 (529) |  |
| - Free State | 9.97 (1 031) | 9.12 (384) | 10.56 (647) |  |
| - Kwa-Zulu Natal | 15.2 (1 571) | 14.32 (603) | 15.8 (968) |  |
| - North West | 10.5 (1 085) | 11.97 (504) | 9.48 (581) |  |
| - Gauteng | 9.97 (1 031) | 11.16 (470) | 9.16 (561) |  |
| - Mpumalanga | 11.8 (1 220) | 12.23 (515) | 11.51 (705) |  |
| - Limpopo | 13.64 (1 410) | 12.99 (547) | 14.09 (863) |  |
| Education level |  |  |  | 0.502 |
| - Primary or less | 26.26 (2 714) | 25.65 (1 080) | 26.67 (1 634) |  |
| - Secondary complete | 64.51 (6 668) | 65.11 (2 741) | 64.1 (3 927) |  |
| - Tertiary | 9.23 (954) | 9.24 (389) | 9.22 (565) |  |
| Wealth index |  |  |  | **0.018** |
| - Quintile 1 (Poorest) | 20.3 (2 098) | 20.45 (861) | 20.19 (1 237) |  |
| - Quintile 2 (Poorer) | 21.55 (2 227) | 22.71 (956) | 20.75 (1 271) |  |
| - Quintile 3 (Middle) | 22.61 (2 337) | 23.06 (971) | 22.3 (1 366) |  |
| - Quintile 4 (Richer) | 19.99 (2 066) | 18.74 (789) | 20.85 (1 277) |  |
| - Quintile 5 (Richest) | 15.56 (1 608) | 15.04 (633) | 15.92 (975) |  |

*Age in years. ‘Categorical variables were tested using Chi-squared, continuous various tested using Wilcoxon.

All self-reported disease conditions were more common in females compared to males (Table 3). While females had a slightly higher prevalence of TB in the last 12 months, though the result was not statistically significant. Other than hypertension, all physically measured disease conditions were significantly more common in females than males. The prevalence of multimorbidity in the sample population was 22.9% (S3 Table).

**Table 3. Prevalence of single disease conditions by sex and method of measurement in South Africa for 2016 (unweighted data).**

| **Disease condition** | **Total %**  **(n/N)** | **Male %**  **(n/N)** | **Female %**  **(n/N)** | ***p*-value** |
| --- | --- | --- | --- | --- |
| **SELF-REPORTED** | | | | |
| Diabetes | 4.5  (459 /10 292) | 3.6  (150 /4 176) | 5.1  (309 /6 116) | **<0.005** |
| Bronchitis / COPD | 1.3  (132 /10 290) | 1.0  (40 /4 177) | 1.5  (92 /6 113) | **<0.005** |
| Heart disease | 3.4  (354 /10 305) | 2.4  (101 /4 183) | 4.1  (253 /6 122) | **0.015** |
| Cholesterol | 2.9  (296 /10 282) | 2.3  (94 /4 167) | 3.3  (202 /6115) | **0.002** |
| Stroke | 1.4  (146 /10 309) | 1.0  (40 /4 186) | 1.7  (106 /6123) | **0.001** |
| TB in last 12 months | 1.3  (138 /10 336) | 1.3  (53 /4 210) | 1.4  (85/6126) | 0.576 |
| **PHYSICALLY MEASURED (BIOMARKER)** | | | | |
| HIV | 19.9  (1 307 /6 584) | 13.8  (346/2 517) | 23.6  (961 /4 067) | **<0.005** |
| Hypertension | 46.2  (3 678/ 7 961) | 45.1  (1 412 /3 130) | 46.9  (2 266 /4 831) | 0.117 |
| Anaemia | 25.9  (1 862/ 7 200) | 17.7  (489/ 2 769) | 31.0  (1 373 /4 431) | **<0.001** |
| Diabetes (HbA1c) | 12.4  (839 /6 763) | 9.3  (241 /2 591) | 14.3  (598 /4 172) | **<0.001** |
| **PHYSICALLY MEASURED (BIOMARKER) AND SELF-REPORTED** | | | | |
| Diabetes (self-report or HbA1c) | 10.06  (1 036 / 10 295) | 7.35  (307/ 4 178) | 11.92  (729 /6 117) | **<0.001** |

## Prevalence of single diseases and multimorbidity

Table 4 shows the weighted prevalence of each included disease condition by sex. Of the self-reported diseases, diabetes had the highest prevalence (4.4%), followed by high cholesterol (3.5%), heart disease (3.1%), COPD or bronchitis (1.4%), stroke (1.4%) and TB in the last 12 months (1.2%). The prevalence of physically measured diseases was higher than that of self-reported diseases. Of the physically measured diseases, hypertension occurred most frequently (45.0%), followed by anaemia (24.7%), HIV (19.6%) and diabetes (11.7%).

Diabetes was the only disease condition included in this study that was both physically measured and self-reported in the questionnaire. The prevalence of physically measured diabetes was more than double that of self-reported diabetes (11.7% versus 4.4%, respectively). This indicates that self-reported diabetes is most likely underreported. When combining the responses of the measured and self-reported diabetes, the composite prevalence was 9.1%. All diseases were more prevalent in females compared to males.

**Table 4. Prevalence of single disease conditions by sex and method of measurement in South Africa for 2016 (weighted data).**

| **Disease condition** | **Total %**  **(95% CI)** | **Male %**  **(95% CI)** | **Female %**  **(95% CI)** |
| --- | --- | --- | --- |
| **SELF-REPORTED** | | | |
| Diabetes | 4.4  (3.9 – 5.0) | 3.7  (3.0 – 4.5) | 5.1  (4.4 – 5.9) |
| Bronchitis / COPD | 1.4  (1.1 – 1.8) | 1.1  (0.8 – 1.6) | 1.7  (1.3 – 2.2) |
| Heart disease | 3.1  (2.7 – 3.5) | 2.3  (1.8 – 2.9) | 3.8  (3.3 – 4.5) |
| Cholesterol | 3.5  (2.9 – 4.2) | 3.0  (2.3 – 3.8) | 4.1  (3.4 – 4.9) |
| Stroke | 1.4  (1.1 – 1.7) | 1.0  (0.7 – 1.5) | 1.7  (1.4 – 2.1) |
| TB in last 12 months | 1.2  (0.9 – 1.5) | 0.9  (0.6 – 1.3) | 1.5  (1.0 – 2.0) |
| **MEASURED (BIOMARKER)** | | | |
| HIV | 19.6  (18.2 – 21.1) | 13.7  (11.8 – 15.8) | 24.5  (22.7 – 26.4) |
| Hypertension | 45.0  (43.1 – 46.9) | 44.1  (41.5 – 46.7) | 45.8  (43.7 – 48.0) |
| Anaemia | 24.7  (23.2 – 26.3) | 16.8  (15.0 – 18.8) | 31.3  (29.2 – 33.5) |
| Diabetes (HbA1c) | 11.7  (10.7 – 12.8) | 9.1  (7.8 – 10.6) | 13.9  (12.6 – 15.4) |
| **MEASURED (BIOMARKER) AND SELF-REPORTED** | | | |
| Diabetes (self-report or HbA1c) | 9.1  (8.4 – 9.9) | 6.9  (5.9 – 7.9) | 11.1  (10.2 – 12.3) |

Note: Biomarker prevalence differed slightly from the DHS report due to the different data cleaning methods and cut-offs employed.

The number of diseases present in one individual ranged from zero to six diseases. The difference in the prevalence in the number of diseases by sex was statistically significant (p<0.001). About 49% of the participants had none of the diseases included in the study, with more males compared to females being “disease-free” (55.8% versus 41.8%, respectively, p<0.001) (Table 5). Less than a third of participants (30.8%) had one disease and there was a difference between males and females (29.4% versus 32.1%, respectively, p=0.0183). Multimorbidity was present in 21% of participants. The prevalence of multimorbidity in females was almost double that of males (26.2% vs 14.8%, respectively) and the difference between the sexes was statistically significant (p<0.001).

**Table 5. Number of diseases in individuals by sex in South Africa for 2016 (weighted data).**

| **Number of diseases** | **Total %**  **(95% CI)** | **Male %**  **(95% CI)** | **Female %**  **(95% CI)** |
| --- | --- | --- | --- |
| No disease | 48.6  (47.0 – 50.1) | 55.8  (53.5 – 58.1) | 41.8  (40.0 – 43.4) |
| 1 disease | 30.8  (29.5 – 32.0) | 29.4  (27.5 – 31.2) | 32.1  (30.6 – 33.7) |
| 2 diseases | 14.1  (13.2 – 15.1) | 10.5  (9.4 – 11.8) | 17.4  (16.2 – 18.7) |
| 3 diseases | 5.2  (4.7 – 5.9) | 3.5  (2.8 – 4.3) | 6.8  (6.0 – 7.8) |
| 4 diseases | 1.1  (0.8 -1.3) | 0.6  (0.4 – 0.9) | 1.4  (1.1 – 1.9) |
| 5 diseases | 0.2  (0.1 – 0.4) | 0.1  (0.1 – 0.3) | 0.4  (0.2 – 0.6) |
| 6 diseases | 0.07  (0.02 – 0.19) | 0.01  (0.01 – 0.04) | 0.01  (0.01 – 0.02) |
| **Multimorbidity (≥ 2 diseases)** | **20.7**  **(19.5 – 21.9)** | **14.8**  **(13.4 – 16.3)** | **26.2**  **(24.7 – 27.7)** |

Multimorbidity prevalence increased with increasing age in both males and females (Fig 1, S3 Table). The prevalence of multimorbidity was consistently higher in females compared to males across the different age groups. Multimorbidity was present at lower levels: 3% for adolescents aged 15 – 19 years and 10% for 20 – 29 year olds. In females, multimorbidity peaked at 47% in the 60 – 69 years olds; whereas in males, it peaked at 40% in the 70 -79 years old. Multimorbidity prevalence dropped slightly in the age group 80 years and over. However, the observed drop is most likely due to uncertainty introduced by a smaller number of people aged 80+ being included in the sample.

**Fig 1. Estimated multimorbidity prevalence by age group and sex in South Africa in 2016.**

People with multimorbidity tended to have an older median age compared to those with no multimorbidity (Fig 2), but this difference was not statistically significant.

**Fig 2. Multimorbidity status by age.**

## Factors associated with multimorbidity

The factors associated with multimorbidity were investigated through a logistic regression (Table 5). For the final model, outliers were dropped and the model was refitted due to its limited ability in predicting multimorbidity in young women with a low BMI (S1 Fig). An interaction term between age and sex was added to the model to improve its fitness.

The final model showed that the odds of being multimorbid increased with age, with the highest odds if the participant was in the 55 – 64 years of age group (OR: 24.910, 95% CI: 14.901 - 41.641, p<0.001), compared to 15 – 24 years old. Younger females (15 – 34 years) had larger odds of being multimorbid compared to males in the same age groups.

The odds of being multimorbid were reduced if an individual had tertiary education (OR: 0.722, 95% CI: 0.537 - 0.970, p=0.031) compared to only having completed primary school education. Those that were employed had a reduced odds of multimorbidity compared to those that were unemployed (OR: 0.813, 95% CI: 0.675 - 0.979, p=0.029). Those that had use alcohol recently also reported a lowered odds compared to those that were not using alcohol (OR: 0.815, 95% CI: 0.686 – 0.968, p=0.02). BMI and current tobacco use were not significant when adjusted for age, sex and other variables. The wealth index was not a predictor of multimorbidity. Additional models can be found in S5 Table.

**Table 5: Factors associated with multimorbidity**

| **Variable** | **Unadjusted**  **Odds ratios**  **(95% CI)** | **Final model (Model 3)**  **Odds ratio**  **(95% CI)** |
| --- | --- | --- |
| Age category *(Reference: 15 – 24 year)* | | |
| - 25 - 34 years | **2.982 (2.407 - 3.695) *** | **3.923 (2.299 - 6.695) *** |
| - 35 - 44 years | **4.861 (3.769 - 6.269) *** | **8.417 (5.101 - 13.890) *** |
| - 45 – 54 years | **7.527 (5.844 - 9.694) *** | **14.165 (8.654 - 23.185) *** |
| - 55 – 64 years | **11.764 (8.837 - 15.662) *** | **24.910 (14.901 - 41.641) *** |
| - 65+ years | **14.181 (10.951 – 18.364) *** | **23.062 (13.719 - 38.766) *** |
| Sex (*Reference: Male)* | **2.038 (1.804 – 2.301) *** | 1.135 (0.831 - 1.551) |
| Age category and sex interaction | | |
| - 15-24#Female | - | **2.734 (1.498 - 4.988) *** |
| - 25-34#Female | - | **1.896 (1.169 - 3.075) *** |
| - 35-44#Female | - | 1.340 (0.842 - 2.132) |
| - 45-54#Female | - | 1.089 (0.676 - 1.755) |
| - 55-64#Female | - | 0.866 (0.558 - 1.345) |
| - 65+#Female | - | **1 (omitted)** |
| Urban *(Reference: Rural)* | **0.817 (0.721 – 0.925) *** | 1.107 (0.901 - 1.360) |
| Education *(Reference: Primary)* | | |
| - Secondary | **0.423 (0.372 -0.480) *** | 0.966 (0.819 - 1.140) |
| - Tertiary | **0.323 (0.251 – 0.414) *** | **0.722 (0.537 - 0.970)** |
| Wealth index (Reference: Poorest) | | |
| - Poorer | 0.995 (0.829 - 1.194) | 1.067 (0.864 - 1.317) |
| - Middle | 1.076 (0.874 - 1.324) | 1.126 (0.867 - 1.464) |
| - Richer | 1.036 (0.845 - 1.270) | 1.034 (0.778 - 1.374) |
| - Richest | 0.901 (0.713 – 1.138) | 0.754 (0.545 - 1.044) |
| Employed *(Reference: Not employed)* | **0.744 (0.643 – 0.861) *** | **0.813 (0.675 - 0.979)** |
| BMI *(Reference: Underweight)* |  |  |
| - Normal weight | 0961 (0.679 – 1.361) | 0.892 (0.609 - 1.309) |
| - Overweight | **1.779 (1.227 – 2.581) *** | 1.033 (0.680 - 1.571) |
| - Obesity group 1 | **2.536 (1.759 – 3.655) *** | 1.213 (0.793 - 1.854) |
| - Obesity group 2 | **2.94 (1.965 – 4.397) *** | 1.340 (0.840 - 2.137) |
| - Obesity group 3 | **3.518 (2.367 – 5.228) *** | 1.527 (0.956 - 2.438) |
| Current alcohol use *(Reference: No current alcohol use)* | **0.571 (0.498 – 0.653) *** | **0.815 (0.686 - 0.968) *** |
| Current tobacco use *(Reference: No current tobacco use)* | **0.704 (0.592 – 0.838)** * | 0.893 (0.710 - 1.122) |

# Discussion

Using the DHS national survey, it was found that one in five South Africans over the age of 15 years was multimorbid. The prevalence of multimorbidity generally increased with age and peaked in the 60 years and older age groups. The prevalence of multimorbidity was higher in females compared to males, but the difference was more striking in younger age groups.

Several other national surveys have been analysed to determine the prevalence of multimorbidity in South Africa.[35-38] The prevalence estimates varied from 2.8%[37] to 63.4%[36], although these studies looked at differing age groups, used varying data collection methods and included different disease conditions. The 2003 World Health Survey which surveyed adults 18 years older found a standardised prevalence of 11.2%[35]. Two waves of the National Income Dynamic Surveys (2008 and 2012) found a low prevalence of 2.7% and 2.8%, respectively[37]. The same 2008 dataset was analysed using different methods but found a similar low prevalence of 4% [38]. Garin, Koyanagi (36) used the 2007/2008 World Health Organization Study on global AGEing (SAGE) and adult health and found a prevalence of 63.4% in adults over the age of 50 years. Most of these studies included self-reported diseases and physically measured hypertension. Self-reported diseases are likely to be underreported as people may be unaware that they have a disease. The DHS physically measured more diseases compared to the National Income Dynamic Surveys and World Health Survey (i.e. HIV, diabetes and anaemia) which may explain its ability to detect higher levels of multimorbidity. The number of disease conditions included in each study also varied. The Garin, Koyanagi (36) study was restricted to adults over the age of 50 years and included a larger number of disease conditions (e.g. depression, cognitive impairment, edentulism and obesity as a disease condition) and therefore reported higher prevalence of multimorbidity. A recent analysis of Wave 2 SAGE (2014/2015) [39] of adults aged 45 years and older, included fewer disease conditions than Garin, Koyanagi (36) (7 vs. 12); reported a multimorbidity prevalence of 21%.

In terms of factors associated with multimorbidity, an increasing age was associated with being multimorbid. This follows trends in reporting in international [32] and the South African literature on multimorbidity . Multimorbidity is often associated with older adults, especially in high income countries [40] due to shifting demographic trends whereby people are living longer, ageing and developing chronic diseases of lifestyle. However, our study had an interesting finding in observing that multimorbidity was present in 10% of young adults between the ages of 20 – 29 years. This is most likely attributed to the high prevalence of single disease such as HIV, anaemia and hypertension in South Africa. HIV is known to affect younger adults in South Africa. A South African national HIV prevalence survey indicated that 7.9% of people (4.8% of males, 10.9% of females) aged 15 – 24 years were HIV positive in 2017 [41]. Also, the prevalence of hypertension in South Africa is thought to be increasing. In young South Africans, hypertension is frequently associated with having a family history of the disease (suggesting a genetic component) and obesity or metabolic syndrome [42,43]. In this study, approximately 44% of people with HIV under 30 years of age, also had hypertension. This has implications for young people in that they will have to be on lifelong treatment for both diseases.

The present study showed that having tertiary education decreased the odds of multimorbidity. This has been noted both locally and internationally [35,36,44]. It was found that being employed decreased the odds of multimorbidity. However, this could also be interpreted to mean that healthier people are more likely to be employed. The wealth index was not significantly associated with multimorbidity. The relationship between wealth and multimorbidity in this study may be unclear as the diseases included may have different patterns according to the individual disease. For example, HIV could be associated with being in a lower wealth quintile, while cardiovascular diseases such as diabetes could be associated with being in a higher wealth quintile. The same argument could be used to explain the findings on BMI. This study indicated that having a high BMI could be associated with multimorbidity but the findings were not significant. High BMI has been identified as associated with multimorbidity in other studies [45]. However, the inclusion of HIV and anaemia could mean that people with lower BMIs were also prone to being multimorbid.

*Limitations*

The current analysis was limited to the data available and disease conditions asked about in the original survey. Additional disease conditions (e.g. cancer) could have been included in the analysis, but the survey in question only asked if the individual had “ever” had the disease. A strength of this study is that disease conditions were only included if the person could have been considered to have the disease at the time of the survey or at a time close to when the study took place. Many studies of multimorbidity include past and current disease conditions without distinction. Had there been included more disease conditions, the prevalence estimates would have most likely be higher.

The study is limited to a simple count of diseases to determine multimorbidity. Studies done with electronic health records or surveyed people specifically for multimorbidity may have taken the severity of diseases into account. Nonetheless, the DHS provides a robust source of data that could be analysed in other LMICs to generate information about multimorbidity where little is still known.

The analysis included self-reported and measured (biomarker) diseases. Self-reported diseases may have been underreported due to participants being unaware that they have a disease. In this study, the prevalence of measured (biomarker) diseases was higher than self-reported diseases. In addition, this study was cross-sectional by nature meaning that we cannot confer temporality.

# Conclusion

This study showed that one in five South Africans, over the age of 15, are managing more than one disease condition. Multimorbidity started in adolescents and increased with age. Females were more frequently affected than males. It was found that having tertiary education and being employed lowered the odds of multimorbidity.

The high prevalence of multimorbidity needs to be addressed in South Africa. This could be done in a twofold manner: (a) by reducing the high prevalence of single diseases such as hypertension and (b) by simultaneously targeting people with existing diseases to reduce their chances of becoming multimorbid. More studies are needed to identify common disease clusters to assist in the endeavour of targeting high risk people. More studies are also needed to determine whether the trends in multimorbidity are changing in the country. For example, to understand whether policies aimed at diseases such as HIV and hypertension have helped to decrease multimorbidity. Also, information is needed on how emerging diseases such as COVID-19 may affect people with multimorbidity in South Africa.

**Contributors**

All authors conceptualised the manuscript. Data analysis was conducted by RAR and AC. RAR drafted the first version of the manuscript. All authors contributed to revising the manuscript and approved the final version.

**Acknowledgements**

We would like to acknowledge the DHS programme for access to the dataset. We would also like to acknowledge Dr Oluwatoyin Awotiwon and Prof Ali Dhansay for their assistance with the inclusion of disease conditions.

**Funding**

The work reported herein was made possible through funding by the Burden of Disease Research Unit at the South African Medical Research Council. RAR conducted this research under the South African Medical Research Council through its Division of Research Capacity Development under the Internship Scholarship Programme from funding received from the South African National Treasury. The content hereof is the sole responsibility of the authors and does not necessarily represent the official views of the South African Medical Research Council or the funders. Grant number: NA.

**Competing interests**

None declared.

**Patient consent for publication**

The anonymised dataset with necessary permissions was obtained from the DHS programme. In addition, ethics clearance was granted by the Biomedical Science Research Ethics Committee of the University of the Western Cape (BM20/5/8).

**Provenance and peer review**

Not commissioned; externally peer reviewed.

**Data availability statement**

The DHS 2016 can be requested from the DHS Programme. Analysis conducted for this paper is available on request.

# REFERENCES

1	Fortin M, Dubois M-F, Hudon C, Soubhi H, Almirall J. Multimorbidity and quality of life: a closer look. Health Qual Life Outcomes. 2007;5:52. doi: 10.1186/1477-7525-5-52

2	Kanesarajah J, Waller M, Whitty JA, Mishra GD. Multimorbidity and quality of life at mid-life: A systematic review of general population studies. Maturitas. 2018;109:53-62. doi: 10.1016/j.maturitas.2017.12.004

3	Lawson KD, Mercer SW, Wyke S, Grieve E, Guthrie B, Watt GCM*, et al.* Double trouble: the impact of multimorbidity and deprivation on preference-weighted health related quality of life a cross sectional analysis of the Scottish Health Survey. Int J Equity Health. 2013;12:67. doi: 10.1186/1475-9276-12-67

4	Makovski TT, Schmitz S, Zeegers MP, Stranges S, van den Akker M. Multimorbidity and quality of life: Systematic literature review and meta-analysis. Ageing Res Rev. 2019;53:100903. doi: <https://doi.org/10.1016/j.arr.2019.04.005>

5	Pati S, Swain S, Knottnerus JA, Metsemakers JF, van den Akker M. Health related quality of life in multimorbidity: a primary-care based study from Odisha, India. Health Qual Life Outcomes. 2019;17:116. doi: 10.1186/s12955-019-1180-3

6	Peters M, Kelly L, Potter CM, Jenkinson C, Gibbons E, Forder J*, et al.* Quality of life and burden of morbidity in primary care users with multimorbidity. Patient Relat Outcome Meas. 2018;9:103. doi: 10.2147/PROM.S148358

7	Loprinzi PD, Addoh O, Joyner C. Multimorbidity, mortality, and physical activity. Chronic Iln. 2016;12:272-80. doi: 10.1177/1742395316644306

8	Nunes BP, Flores TR, Mielke GI, Thume E, Facchini LA. Multimorbidity and mortality in older adults: a systematic review and meta-analysis. Arch Gerontol Geriatr. 2016;67:130-8. doi: <http://dx.doi.org/10.1016/j.archger.2016.07.008>

9	Wei MY, Mukamal KJ. Multimorbidity, mortality, and long-term physical functioning in 3 prospective cohorts of community-dwelling adults. Am J Epidemiol. 2018;187:103-12. doi: <https://doi.org/10.1093/aje/kwx198>

10	Frølich A, Ghith N, Schiøtz M, Jacobsen R, Stockmarr A. Multimorbidity, healthcare utilization and socioeconomic status: A register-based study in Denmark. PLoS One. 2019;14:e0214183. doi: 10.1371/journal.pone.0214183

11	Lehnert T, Heider D, Leicht H, Heinrich S, Corrieri S, Luppa M*, et al.* Review: Health Care Utilization and Costs of Elderly Persons With Multiple Chronic Conditions. Med Care Res Rev. 2011;68:387-420. doi: 10.1177/1077558711399580

12	Palladino R, Tayu Lee J, Ashworth M, Triassi M, Millett C. Associations between multimorbidity, healthcare utilisation and health status: evidence from 16 European countries. Age Ageing. 2016;45:431-5. doi: 10.1093/ageing/afw044

13	Sum G, Salisbury C, Koh GC, Atun R, Oldenburg B, McPake B*, et al.* Implications of multimorbidity patterns on health care utilisation and quality of life in middle-income countries: cross-sectional analysis. J Glob Health. 2019;9:020413. doi: 10.7189/jogh.09.020413

14	Zulman DM, Pal Chee C, Wagner TH, Yoon J, Cohen DM, Holmes TH*, et al.* Multimorbidity and healthcare utilisation among high-cost patients in the US Veterans Affairs Health Care System. BMJ Open. 2015;5:e007771. doi: 10.1136/bmjopen-2015-007771

15	Xu X, Mishra GD, Jones M. Mapping the global research landscape and knowledge gaps on multimorbidity: a bibliometric study. J Glob Health. 2017;7:010414-. doi: 10.7189/jogh.07.010414

16	The World Bank. Data: South Africa. Internet. 2020. <http://data.worldbank.org/country/south-africa> (Accessed: 2 April 2021)

17	Pillay-van Wyk V, Msemburi W, Laubscher R, Dorrington RE, Groenewald P, Glass T*, et al.* Mortality trends and differentials in South Africa from 1997 to 2012: second National Burden of Disease Study. Lancet Glob Health. 2016;4:e642-e53. doi: <https://doi.org/10.1016/S2214-109X(16)30113-9>

18	Roomaney RA, van Wyk B, Turawa EB, Pillay-van Wyk V. Multimorbidity in South Africa: a systematic review of prevalence studies. BMJ Open. 2021;11:e048676. doi: 10.1136/bmjopen-2021-048676

19	Short Fabic M, Choi Y, Bird S. A systematic review of Demographic and Health Surveys: data availability and utilization for research. Bull World Health Organ. 2012;90:604-12. doi: 10.2471/BLT.11.095513

20	National Department of Health, Statistics South Africa, South African Medical Research Council, and ICF. South Africa Demographic and Health Survey 2016. Pretoria, South Africa, and Rockville, Maryland, USA: NDoH, Stats SA, SAMRC, and ICF, 2019. <https://dhsprogram.com/pubs/pdf/FR337/FR337.pdf> (Accessed: 23 December 2020)

21	The International Expert Committee. International Expert Committee Report on the Role of the A1C Assay in the Diagnosis of Diabetes. Diabetes Care. 2009;32:1327-34. doi: 10.2337/dc09-9033

22	World Health Organization. Definition, diagnosis and classification of diabetes mellitus and its complications: report of a WHO consultation. Part 1, Diagnosis and classification of diabetes mellitus. World Health Organization, 1999. <https://apps.who.int/iris/handle/10665/66040> (Accessed: 30 July 2021)

23	ICF. Demographic and Health Surveys Standard Recode Manual for DHS7. Rockville, Maryland, U.S.A.: ICF: The Demographic and Health Surveys Program.; 2018.

24	Chobanian AV, Bakris GL, Black HR, Cushman WC, Green LA, Izzo Jr JL*, et al.* The seventh report of the joint national committee on prevention, detection, evaluation, and treatment of high blood pressure: the JNC 7 report. JAMA. 2003;289:2560-71. doi: 10.1001/jama.289.19.2560.

25	Pillay van Wyk V, et al. Estimated attributable burden from high fasting plasma glucose for South Africa: 2000, 2006 and 2012 S Afr Med J. 2021;Forthcoming. doi: NA

26	Nojilana B, et al. Estimated attributable burden from hypertenion for South Africa: 2000, 2006 and 2012 S Afr Med J. 2021;Forthcoming. doi: NA

27	Bradshaw D, et al. Estimating the changing disease burden attributable to high body mass index in South Africa in 2000, 2006 and 2012. S Afr Med J. 2021;Forthcoming. doi: NA

28	Statistics South Africa. Mid-year population estimates 2016. 2016. <https://www.statssa.gov.za/publications/P0302/P03022016.pdf> (Accessed: 8 July 2021)

29	Huntley AL, Johnson R, Purdy S, Valderas JM, Salisbury C. Measures of multimorbidity and morbidity burden for use in primary care and community settings: a systematic review and guide. Ann Fam Med. 2012;10:134-41. doi: 10.1370/afm.1363.

30	Sinnige J, Braspenning J, Schellevis F, Stirbu-Wagner I, Westert G, Korevaar J. The prevalence of disease clusters in older adults with multiple chronic diseases–a systematic literature review. PLoS One. 2013;8:e79641. doi: 10.1371/journal.pone.0079641.t001

31	Hosmer Jr DW, Lemeshow S, Sturdivant RX. Applied logistic regression: John Wiley & Sons; 2013.

32	Xu X, Mishra GD, Jones M. Evidence on multimorbidity from definition to intervention: An overview of systematic reviews. Ageing Res Rev. 2017;37:53-68. doi: <https://doi.org/10.1016/j.arr.2017.05.003>

33	StataCorp. Stata User's Guide: Release 17. 2021. <https://www.stata.com/manuals/u.pdf> (Accessed: 23 July 2021)

34	Pregibon D. Logistic regression diagnostics. The annals of statistics. 1981;9:705-24. doi: 10.1214/aos/1176345513

35	Afshar S, Roderick PJ, Kowal P, Dimitrov BD, Hill AG. Multimorbidity and the inequalities of global ageing: a cross-sectional study of 28 countries using the World Health Surveys. BMC Public Health. 2015;15:776. doi: <https://doi.org/10.1186/s12889-015-2008-7>

36	Garin N, Koyanagi A, Chatterji S, Tyrovolas S, Olaya B, Leonardi M*, et al.* Global Multimorbidity Patterns: A Cross-Sectional, Population-Based, Multi-Country Study. J Gerontol A Biol Sci Med Sci. 2016;71:205-14. doi: 10.1016/j.seizure.2019.06.018

37	Weimann A, Dai D, Oni T. A cross-sectional and spatial analysis of the prevalence of multimorbidity and its association with socioeconomic disadvantage in South Africa: A comparison between 2008 and 2012. Soc Sci Med. 2016;163:144-56. doi: <https://doi.org/10.1016/j.socscimed.2016.06.055>

38	Alaba O, Chola L. The social determinants of multimorbidity in South Africa. Int J Equity Health. 2013;12:1. doi: <https://doi.org/10.1186/1475-9276-12-63>

39	Chidumwa G, Maposa I, Corso B, Minicuci N, Kowal P, Micklesfield LK*, et al.* Identifying co-occurrence and clustering of chronic diseases using latent class analysis: cross-sectional findings from SAGE South Africa Wave 2. BMJ Open. 2021;11:e041604. doi: 10.1136/bmjopen-2020-041604

40	Ofori-Asenso R, Chin KL, Curtis AJ, Zomer E, Zoungas S, Liew D. Recent patterns of multimorbidity among older adults in high-income countries. Popul Health Manag. 2019;22:127-37. doi: 10.1089/pop.2018.0069

41	Simbayi L, Zuma K, Moyo S, Marinda E, Mabaso M, Ramlagan S*, et al.* South African National HIV Prevalence, Incidence, Behaviour and Communication Survey, 2017. Cape Town: HSRC Press, 2019 978-0-7969-2444-5. <https://www.hsrcpress.ac.za/books/south-african-national-hiv-prevalence-incidence-behaviour-and-communication-survey-2017> (Accessed: 23 December 2020)

42	Mangena P, Saban S, Hlabyago KE, Rayner B. An approach to the young hypertensive patient. S Afr Med J. 2016;106:36-8. doi: 10.7196/SAMJ.2016.v106i1.10329

43	Jones ESW, Esack I, Mangena P, Rayner BL. Hypertension in adolescents and young adults referred to a tertiary hypertension clinic in Cape Town, South Africa. Medicine. 2020;99:e23137-e. doi: 10.1097/MD.0000000000023137

44	Johnson-Lawrence V, Zajacova A, Sneed R. Education, race/ethnicity, and multimorbidity among adults aged 30–64 in the National Health Interview Survey. SSM Popul Health. 2017;3:366-72. doi: <https://doi.org/10.1016/j.ssmph.2017.03.007>

45	Agrawal S, Agrawal PK. Association Between Body Mass index and Prevalence of Multimorbidity in Low-and Middle-income Countries: A Cross-Sectional Study. Int J Med Public Health. 2016;6:73-83. doi: 10.5530/ijmedph.2016.2.5
