# Supplementary material for: One in five South Africans are multimorbid: An analysis of the 2016 demographic and health survey
Source: PLoS One. 2022 May 26;17(5):e0269081. doi: 10.1371/journal.pone.0269081 (PMC9135225; doi:10.1371/journal.pone.0269081)
Supplement: Attachment — Submitted filename: Response to reviewer comments_2nd revision.docx [file pone.0269081.s004.docx]

**Manuscript ID PONE-D-21-34287R1**

**Response to Reviewers**

Dear Dr Pathak,

Thank you for once again providing us with the opportunity to submit a revised draft of the manuscript “One in five South Africans are multimorbid: An analysis of the 2016 Demographic and Health Survey” to PLOS ONE.

We thank Reviewer 2 for the thorough review and for suggesting that data be added to the supplementary appendix. We have added an extra graph to the appendix and applied all the changes the reviewer suggested. Please see our responses to the reviewer comments below.

| **Comment** | **Response** | **Page & line number** |
| --- | --- | --- |
| **Editor comments (Dr Mona Pathak)** | | |
| 1. Please review your reference list to ensure that it is complete and correct.   If you have cited papers that have been retracted, please include the rationale for doing so in the manuscript text, or remove these references and replace them with relevant current references. If you need to cite a retracted article, indicate the article’s retracted status in the References list and also include a citation and full reference for the retraction notice.  Any changes to the reference list should be mentioned in the rebuttal letter that accompanies your revised manuscript. | References were checked for completeness and errors.  No retracted papers are cited. | N/A. |
| **Reviewer 2** | | |
| 1. Line 246: delete the sub-title factors associated with multimorbidity | Done. The sub-title was deleted. | Line 221, Page 12. |
| 2. Line 267: Cut the sentence "Three models were constructed for logistic regression with multimorbidity as the dependent variable and paste it at line 257 before Model 1 | Done. The sentence was moved to where the reviewer suggested. | Line 227-228, Page 12. |
| 3. Line 284: the sentence whiles females had a slightly higher prevalence of TB in the last 12 months, though...... is not complete. Kindly check and complete it. | Done. The sentence was changed to:  *Females had a slightly higher prevalence of TB in the last 12 months compared to males, however, the difference was not statistically significant.* | Line 256 – 257, Page 14. |
| 4. Line 309: delete "disease" after six | Done. Word was deleted. | Line 282, Page 16. |
| 5.Line 357: change over the age 15 years to aged 15years or above | Done. Wording changed as suggested. | Line 330-331, Page 20. |
| 6. Line 362-364: which group of people did the study focused on? adult men, women or both? | Added text “in females 15 years or above”. | Line 337, Page 21. |
| 7. Line 333: if the data is available, kindly show it as an appendix to erase doubts among readers | We thank the reviewer for insisting on adding the data. Upon relooking at the data, the quoted prevalence was for the unweighted whole population and not for those under 30 years.  We have added the corrected weighted prevalence for those under 30 years (32%) and added a supporting figure in the supplementary appendix (Fig S3). | Line 373, Page 22.  Figure S3 in appendix added. |
| 8.Line 440: change over the age 15 years to 15 years or above | Done. Wording changed as suggested. | Line 420, Page 24. |
